# Xylanase and *Bacillus subtilis* PB6 modulate microbiota and short-chain fatty acid profiles in broilers under necrotic enteritis-challenge

**DOI:** 10.1016/j.psj.2025.106330

**Published:** 2025-12-22

**Authors:** Most Khairunnesa, Alip Kumar, Shu-Biao Wu, Mingan Choct, Yadav Sharma Bajagai, Kosar Gharib-Naseri

**Affiliations:** a School of Environmental and Rural Science, University of New England, Armidale, NSW 2351, Australia; b Institute for Future Farming Systems, Central Queensland University, Rockhampton, QLD, 4702, Australia

**Keywords:** Xylanase, *Bacillus subtilis*, Short-chain fatty acids, Gene expression, Broiler

## Abstract

Necrotic enteritis (**NE**) is a major poultry disease affecting profitability. Managing NE has become harder due to restrictions on in-feed antibiotics, which traditionally support gut health and production in broilers. To address this, a study was conducted to evaluate the effect of xylanase (**Xy**) and *Bacillus subtilis* (**Pb**) supplementation in a corn-soybean-based diet on the intestinal health of broiler chickens under NE challenge. A total of 630-d-old mixed-sex Cobb 500 broiler chicks were assigned to a 2 × 2 + 1 factorial design, giving five treatments: NE challenge without additives (**CC**); NE challenge with Xy (0.03%) (**Xy**); NE challenge with Pb (0.05%) (**Pb**); NE challenge with Xy (0.03%) and Pb (0.05%) (**Xy+Pb**); and non-challenge birds without additives (**NC**). NE challenge significantly increased serum fluorescein isothiocyanate dextran (**FITC-d**; *P* < 0.05), as well as ileal lactate, succinate, and total short-chain fatty acids (**SCFA**) concentrations (*P* < 0.05), while downregulating immunoglobulin A (***IgA***) and mucin 2 (***MUC2***) (*P* < 0.05). Pairwise permutational multivariate analysis of variance (**PERMANOVA**) showed distinct microbial profiles between the NC and CC groups (*P* < 0.05; unweighted and weighted UniFrac). Supplementation of Pb altered beta diversity compared with the CC, showing greater distances (weighted UniFrac; *P* < 0.05). It also shifted the relative abundances of Ruminococcaceae and *Faecalibacterium* towards NC levels, with no significant differences from either CC or NC groups (*P* > 0.05). Dietary Pb increased (*P* < 0.05) ileal lactate and total SCFA concentrations, irrespective of NE challenge. Dietary Xy significantly reduced Lachnospiraceae UCG 010 relative to CC birds (*P* < 0.05). It also showed tendencies towards lowering caecal propionate, iso-butyrate, and iso-valeric concentrations, and decreasing interferon gamma (***IFN*-*γ***) gene expression (*P* < 0.1). Overall, these results indicate that both Xy and Pb may help mitigate the adverse effects of NE by modulating gut microbiota and, thus, gut health. However, Pb showed greater and more consistent effects on SCFA production, microbiota diversity, and beneficial bacteria populations, highlighting its greater efficacy under NE challenge.

## Introduction

Necrotic enteritis (**NE**) is a highly prevalent disease in broilers caused by pathogenic strains of *Clostridium perfringens*, often triggered by predisposing factors such as coccidia infection, high-protein diets, fishmeal, and non-starch polysaccharides (Engström et al., 2003; Moore, 2016). This disease is responsible for substantial economic losses, estimated at over USD 6 billion annually in the global poultry industry (Moore, 2016; Wade and Keyburn, 2015)*.* Coccidiosis, particularly from *Eimeria* species, contributes to damage of intestinal epithelial cells and increases mucus production, thereby facilitating *C. perfringens* colonization and overgrowth (Collier et al., 2008). Previous studies have shown that NE disrupts gut microbial balance, alters short-chain fatty acids (**SCFA**) profiles (Gharib-Naseri et al., 2019; Stanley et al., 2014) and increases gut permeability (Kumar et al., 2021). Furthermore, NE has been linked to the downregulation of critical genes involved in mucosal defense, including mucin 2 (***MUC2***), tight junction proteins (claudin 5, ***CLDN5***; occludin, ***OCLDN*;** and tight junction protein 1, ***TJP1***), immunoglobulin M (***IgM***), and interleukin-6 (***IL6***), while upregulating pro-inflammatory genes such as interferon-gamma (***IFNγ***) (Dao et al., 2022; Gharib-Naseri et al., 2021; Keerqin et al., 2021).

Subtherapeutic in-feed antibiotics have long been used as a management practice to manage NE in poultry (Ningsih et al., 2023). However, concerns over antimicrobial resistance and the transfer of resistance genes to humans (He et al., 2020; Martin et al., 2015; Yang et al., 2022; Zhang et al., 2015) have led to regulatory restrictions or bans on in-feed antibiotics use in many countries. Consequently, there is an urgent need for alternative gut health strategies to maintain the productivity of poultry. Among these alternatives, exogenous enzymes, especially xylanase, have emerged as promising alternatives due to their potential in nutritional and health benefits (DoskoviĆ et al., 2013). Xylanase breaks down non-starch polysaccharides in wheat (Zhang et al., 2014) and corn-based diets (Masey-O'Neill et al., 2014), producing xylooligosaccharides that can act as prebiotics (Craig et al., 2020). The oligosaccharides promote the growth of beneficial gut bacteria such as *Lactobacillus* spp. and *Bifidobacterium* spp., while inhibiting *C. perfringens* (Lin et al., 2016). Additionally, xylanase has been reported to improve intestinal mucosal integrity (Liu et al., 2012), promote *Lactobacilli* growth (Singh and Kim, 2021; Van Hoeck et al., 2021; Zhang et al., 2025), and upregulate expression of nutrient transporter genes (Hosseini et al., 2017). Despite the reported benefits, its effects have not been consistent across studies. For example, in a wheat-based diet, xylanase, along with beta-glucanase, did not enhance the expression of nutrient transporter genes or gut integrity under *Eimeria*-challenged boilers (Daneshmand et al., 2023). In contrast, xylanase supplementation increased the abundance of *Bifidobacterium, Lactobacillus*, and Enterobacteriaceae, as well as butyric acid in the caeca of birds fed a wheat-based diet compared to a corn-based diet under NE challenge (Kim et al., 2022). Under normal conditions, the type of cereal also matters; with wheat, xylanase increased the abundance of *Lactobacillus, Bifidobacterium*, and several butyrate-producers in birds, while in corn, the shifts were modest (Wang et al., 2021a). Overall, the efficacy of xylanase seems to depend on both the diet composition and health status of birds, warranting further research to clarify its role and optimize its application under such dietary and disease-challenge conditions.

Another promising feed additive is *Bacillus subtilis*, a spore-forming probiotic known for its heat stability and ability to survive feed processing and gut transit (Ramlucken et al., 2020). This microorganism has been shown to enhance microbial balance, immunity, and nutrient utilization, thereby supporting overall gut health in broilers (Chaudhari et al., 2020; Fazelnia et al., 2021; Wang et al., 2021b; Yaqoob et al., 2022). Specifically, the PB6 strain of *B. subtilis* has shown to improve growth performance, intestinal barrier function, caecal SCFA levels, and reduce intestinal inflammation in NE-challenged birds (Aljumaah et al., 2020; Jayaraman et al., 2013; Liu et al., 2021c). Conversely, Aljumaah et al. (2020) reported a significant shift in beta diversity in birds fed Pb under NE. In contrast, Liu et al. (2021c) observed no difference in beta diversity. Liu et al. (2025) observed a higher abundance of proteolytic and butyrate-producing bacteria, as well as a higher butyric acid concentration, in piglets fed a high-protein diet supplemented with PB6 strain, in the absence of a disease challenge. On the other hand, high concentration of acetic acid was reported in NE-challenged broilers fed the PB6 strain (Aljumaah et al., 2020). These contradictory findings with Pb make it challenging to draw clear conclusions and underscore the need for further research. Evidence suggests that combining enzymes and probiotics in chickens may exert complementary effects, thereby enhancing gut health more effectively than either additive alone. In broilers, co-supplementation has been reported to enhance enzyme activity and microbial profiles (Wang et al., 2021b), improve nutrient digestibility (Singh et al., 2019), stimulate immune response (Seidavi et al., 2017), increase caecal SCFA concentrations (Gao et al., 2022; Luo et al., 2023), and upregulate intestinal barrier genes (*claudin-1, ZO-1*, and *mucin-2*), elevate *Lactobacilli* counts (Agboola et al., 2021) in non-challenged conditions. Similarly, in fish, enzyme-probiotic co-supplementation has shown to improve intestinal histomorphology (Adeoye et al., 2016) and increase the abundance of beneficial microbes such as lactic acid bacteria and *Bacillus* species (Maas et al., 2021). Based on this rationale, the current study hypothesizes that dietary supplementation with xylanase (0.03%) and *B. subtilis* PB6 (0.05%), either individually or in combination, improves gut integrity and SCFA profile, modulates caecal microbiota composition, and regulates the intestinal gene expression in broilers challenged with subclinical NE. Therefore, the objective of the current study was to investigate the potential of xylanase and *B. subtilis* PB6, either alone or in combination, to enhance overall gut health and elucidate their underlying mode of action in broilers challenged with SNE.

## Materials and methods

### Ethics statement

The management and handling procedures for the birds followed the guidelines approved by the Animal Ethics Committee at the University of New England, Australia (ARA22-005). All procedures were conducted in accordance with the Australian Bureau of Animal Health's accredited guidelines for the ethical use and care of laboratory animals (NHMRC, 2013).

### Experimental birds, design, and diets

This study continued a previous study by Khairunnesa et al. (2025), which reported birds' performance, liveability, gut histology, and intestinal lesions results. A total of 630 mixed-sex Cobb 500-d-old broiler chicks were used in this study. The birds were obtained from a commercial hatchery (Baiada Hatchery in Tamworth, NSW, Australia). Upon arrival, the chicks were randomly allocated to 45-floor pens in a completely randomized design, following a 2 × 2 factorial arrangement plus one additional treatment (thus 2 × 2 + 1), resulting in five experimental treatments. Initial pen weights were balanced across treatments to avoid significant differences at the start of the trial. Each treatment consisted of nine replicate pens, with 14 birds per pen. The treatments were: CC (NE challenged without additives), Xy (NE challenged with Xy at 0.03%), Pb (NE challenged with Pb at 0.05%), Xy+Pb (NE challenged with Xy at 0.03% and Pb at 0.05%), and NC (non-challenged birds with no additives) (Table 1). Softwood shavings were used as bedding material, and each pen was equipped with a tube feeder and three nipple drinkers, providing ad libitum access to feed and water. Environmental conditions, including temperature, humidity, and lighting, were maintained according to Cobb 500 management guidelines (Cobb 500, 2022b).

**Table 1:** Treatments applied in this study.

1Treatments	Feed additives		Inclusion level, %	2Necrotic enteritis challenge
	Xylanase (Xy)	*Bacillus subtilis* PB6 (Pb)		
CC	No	No	-	Challenged
Pb	No	Yes	0.05	Challenged
Xy	Yes	No	0.03	Challenged
Xy+Pb	Yes	Yes	0.03+0.05	Challenged
NC	No	No	-	Non-challenged

1 CC: challenged control, Xy: challenged control+ xylanase (0.03%), Pb: challenged control+ *B. subtilis* PB6 (0.05%), Xy + Pb: challenged control+ xylanase (0.03%) +*B. subtilis* PB6 (0.05%), NC: non-challenged control.

2 NE-challenged birds were orally gavaged with *Eimeria* spp. on d9 and *Clostridium perfringens* on d14 and 15.

Experimental diets were formulated based on corn and soybean meal with added phytase, at 500 FTU/Kg (100 g/t), according to the feeding standards and nutrient specifications for Cobb 500 broilers (Cobb 500, 2022a). Feed ingredients were analyzed using near-infrared spectroscopy before formulation of the diets (AminoNIR, Evonik Amino Prox, Essen, Germany). Basal diets were formulated using sand as filler. In the additive groups, sand was partially replaced with additives: 0.03% sand was replaced with xylanase in the Xy group, 0.05% with *B. subtilis* in the Pb group, and a combined amount of Xy and Pb in the combination group. Additives were blended into the feed during the mixing step of feed preparation prior to the pelleting. Birds were provided *ad libitum* access to diets, which were offered as crumbles during the starter phase (d0-8), as pellets during the grower (d9-19), and finisher (d20-35) phases. The detailed composition for each phase and nutrient contents are shown in Table 2, and the analyzed nutrients of the feed are presented in supplementary Table S1.

**Table 2:** Diet composition (as-fed basis, %), and calculated nutrients.

Ingredients (%)	Starter (d0-8)	Grower (d9-19)	Finisher (d20-35)
Corn	58.1	62.5	67.2
Soybean meal	36.6	31.6	26.9
Canola oil	1.55	1.80	2.10
Dicalcium phosphate	1.44	0.97	0.90
Limestone	0.96	0.75	0.64
DL-methionine	0.34	0.32	0.30
Salt	0.26	0.26	0.24
L-lysine HCl 78.4	0.21	0.24	0.28
L-threonine	0.15	0.11	0.12
Choline Cl 60%	0.09	0.12	0.14
1Mineral Premix	0.08	0.08	0.08
2Vitamin Premix	0.08	0.08	0.08
Sodium bicarbonate	0.03	0.03	0.03
3Phytase	0.01	0.01	0.01
4Sand	0.08	1.17	0.98
5**Calculated nutrients (%, otherwise as indicated)**			
Dry Matter	89.0	89.0	88.8
AME (kcal/kg)	2,950	3,000	3,075
Crude protein	22.7	20.7	18.9
Crude fat	3.90	4.22	4.61
Crude fiber	3.24	3.12	3.03
Digestible Arg	1.34	1.20	1.07
Digestible Lys	1.26	1.16	1.08
Digestible Met	0.61	0.57	0.54
Digestible Meth+Cyst	0.94	0.88	0.83
Digestible Trp	0.28	0.26	0.23
Digestible Iso	0.87	0.78	0.70
Digestible Thr	0.90	0.79	0.74
Digestible Val	0.93	0.84	0.77
Calcium	0.96	0.80	0.74
Available phosphorus	0.54	0.40	0.37
Sodium	0.19	0.18	0.17
Potassium	1.10	1.01	0.93
Chloride	0.25	0.26	0.26
Choline mg/kg	1,716	1,700	1,704
Linoleic 18:2	1.50	1.59	1.70

AME, apparent metabolizable energy.

1 Trace mineral concentrate supplied per kilogram of diet: Cu (sulfate), 16 mg; Fe (sulfate), 40 mg; I (iodide), 1.25 mg; Se (selenate), 0.3 mg; Mn (sulfate and oxide), 120 mg; Zn (sulfate and oxide), 100 mg; cereal-based carrier, 128 mg; mineral oil, 3.75 mg.

2 Vitamin premix per kg diet:vitamin A, 12 MIU; vitamin D, 5 MIU; vitamin E, 75 mg; vitamin K, 3 mg; nicotinic acid, 55 mg; pantothenic acid, 13 mg; folic acid, 2 mg; riboflavin, 8 mg; cyanocobalamin, 0.016 mg; biotin, 0.25 mg; pyridoxine, 5 mg; thiamine, 3 mg; antioxidant, 50 mg.

3 Phytase: Quantum Blue 5G, AB Vista, 500 FTU/kg of diet.

4 Sand was replaced with the required amount of xylanase and *B. subtilis* PB6 and added to the top.

5 Nutrient contents were measured using near-infrared spectroscopy (NIRS, Evonik Amino Prox, Germany).

### Feed additives

The additives tested were xylanase (Xygest™ HT) and *B. subtilis* PB6 (CLOSTAT™), provided by Kemin Animal Health and Nutrition, Singapore. Xygest™ HT is a thermostable, monocomponent xylanase enzyme derived from *Thermopolyspora flexuosa* and expressed in *Pichia pastoris*. As a member of the GH11 family of beta-1,4 endo-xylanases, it is designed to break down dietary fibre, thereby enhancing energy utilization. With a minimum activity of 3,000,000 U/g on a cornstarch-based carrier, Xygest™ HT is recommended at 10 g/t, providing 30,000 U/kg of feed. ClOSTAT™ contains PB6, a unique strain of *B. subtilis*, a naturally occurring spore-forming bacterium that maintains its stability during feed processing. It is included at a concentration of 2.2 × 10⁸ CFU/g of product, equivalent to 1 × 10⁸ CFU/kg of feed.

### NE challenge

The NE challenge protocol followed the methods described by Wu et al. (2014) and Rodgers et al. (2015). Briefly, on d9, birds were orally gavaged with 1 mL of a mixed *Eimeria* spp. suspension containing 5000 oocysts each of *Eimeria acervulina* and *E. maxima* and 2500 oocysts of *E. brunetti* (Eimeria Pty Ltd., Ringwood, VIC, Australia). On d14 and 15, the same birds were orally gavaged with 1 mL *C. perfringens* (EHE-NE18) at approximately 1 × 10^8^ CFU (CSIRO Livestock, Geelong, VIC, Australia). Concurrently, the non-challenged control birds received a sham challenge: 1 mL of phosphate-buffered saline (**PBS**) on d9 and 1 mL of sterile thioglycolate broth on d14 and 15.

### Bird sex identification, FITC-d inoculation, and sample collection

To confirm the presence of one male and one female in each pen for sampling, feather samples were collected for bird sexing. For this purpose, six birds from each replicated pen were selected and tagged with different-coloured leg bands. Two feathers from each bird were collected and squeezed the feather follicles in a 2 mL tube, and followed the DNA extraction and PCR analysis method previously described by England et al. (2021). High-resolution melting curve analysis was used to confirm sex based on melting profiles. After sex confirmation in the lab, leg band tags were removed, and the birds’ feathers were coloured with blue (male) and pink (female) to identify sex. On d16, one male and one female from each pen previously confirmed were selected, weighed, and orally gavaged with 1 mL of FITC-d (average molecular weight: 4000, Sigma-Aldrich Co., St. Louis, MO, USA), at a dose of 4.17 mg/kg average body weight. Approximately 2.5 hours after inoculation, the birds were stunned using an electric stunner (JF Poultry Equipment, Weltevreden Park, South Africa) and then euthanized by cervical dislocation. Blood samples were collected from the jugular vein into a clot activator vacutainer and were kept at room temperature for 3 hours to clot. Samples were then centrifuged at 3,000 × *g* for 10 minutes, and serum was separated and stored at −20°C until further analysis. Caecal contents from each of the 2 sample birds per pen were separately collected for microbiota analysis, and the rest of the caecal samples were pooled for SCFA analysis. Similarly, ileal samples were collected from the same 2 sample birds in each pen and pooled before analysis. All the samples were stored at −20°C until further processing. Furthermore, approximately 1 cm of jejunal tissue was collected in a 2 mL tube containing RNA later and stored at −20°C until further use.

### Analysis of FITC-d

Serum FITC-d concentrations were measured using a multi-mode microplate reader (Spectra Max M2e, Molecular Devices, San Jose, CA, USA). Serum samples were diluted with PBS in a 1:1 ratio prior to analysis. The fluorescence was determined using excitation at 485 nm and emission at 528 nm. The FITC-d concentrations were calculated based on a standard curve, which was constructed using known concentrations of FITC-d per mL. Results were expressed in µg/mL and were adjusted for the individual body weight of the birds.

### Analysis of caecal and ileal contents for SCFA

Ileal and caecal pooled samples were analyzed following the method described by Jensen et al. (1995). In short, approximately 0.8 g of caecal and 1.5 g of ileal pooled samples (stored at −20°C) were weighed separately into different centrifuge tubes and kept on ice. To each tube, 1 mL of a standard solution (0.01 M methyl butyric acid) was added and vortexed briefly for proper mixing. The mixed solution was centrifuged at 15,000 × *g* for 20 minutes at 5°C. After centrifugation, 1 mL of the supernatant was carefully transferred into 8 mL vials. Subsequently, 0.5 mL of concentrated HCl (36%) and 2.5 mL of diethyl ether were added to the vial and vortexed, followed by centrifugation at 3,000 × *g* for 15 min at 5°C. To these vials, 40 μL of N‑tert-butyldimethylsilyl-N-methyl trifluoroacetamide was added and vortexed gently for proper mixing. Subsequently, the vials were placed in a heating block at 80°C for 20 minutes. After heating, the GC vial lids were tightened, and the vials were left at room temperature for 48 hours before analysis. Finally, the 2 mL GC vials were analyzed using a gas chromatograph (Varian CP3400 CX, Varian Analytical Instruments, Palo Alto, CA, USA). The concentrations of both ileal and caecal SCFAs were expressed as µmol/g of digesta samples.

### RNA extraction and cDNA synthesis

RNA extraction from jejunal tissue was performed using the Bioline ISOLATE II RNA Mini Kit (Bioline Meridian Bioscience, Australia). Approximately 25-35 mg of tissue was placed in a 2 mL Eppendorf tube with a 3 mm metal bead. To this, 350 µL of Buffer RLY-β-ME (350 µL lysis buffer RLY + 3.5 µL β-ME) was added, and the sample was homogenized with a Tissuelyser II (Qiagen, Hilden, Germany). The homogenate was transferred to a 2 mL ISOLATE II filter placed on a 2 mL collection tube and centrifuged at 11,000 × *g* for 1 min. Following centrifugation, 350 µL of 70% ethanol was added, mixed thoroughly, and the lysate was centrifuged at 11,000 × *g* for 30 seconds through an ISOLATE II mini-column. The column was then placed into a new 2 mL collection tube, with 350 µL of Membrane Desalting Buffer added, and centrifuged at 11,000 × *g* for 1 min to dry the membrane. To remove genomic DNA, 95 µL of a DNase I reaction mixture (10 µL DNase *I* + 90 µL RDN buffer) was applied directly to the silica membrane and incubated at room temperature for 15 min. The samples were washed twice with wash buffers (200 µL RW1 and 600 µL RW2) and centrifuged at 11,000 × *g* after each wash, with a final wash of 250 µL RW2 and centrifugation at 11,000 × *g* for 2 min. Subsequently, 100 µL of nuclease-free water was added to the silica membrane, followed by centrifugation at 11,000 × *g* for 1 min. The column was discarded, and the RNA was stored at −20°C. RNA quantity and purity were measured using a Nanodrop ND-8000 spectrophotometer (Thermo Fisher Scientific, Waltham, MA, USA). RNA integrity was assessed with an Agilent 2100 Bioanalyzer using RNA 6000 Nano Kit (Agilent Technologies, Inc., Waldron, Germany), aiming for a 260/230 ratio > 1.8, a 260/280 ratio between 2.0 and 2.2, and an RNA Integrity Number (RIN) > 7.0.

For reverse transcription, 1 µg of RNA was incubated with 2 µL of 7 gDNA Wipeout Buffer at 42°C for 2 minutes to eliminate genomic DNA. The reaction mixture, including 1 µL QuantiTect Reverse Transcriptase, 4 µL of 7 × Quantiscript RT Buffer, and 1 µL RT Primer Mix, was incubated at 42°C for 15 min and then at 95°C for 3 min to convert RNA to cDNA using the Rotorgene 6000 real-time PCR system (Corbett, Sydney, Australia). The cDNA was diluted fivefold in nuclease-free water and stored at −20°C.

### Real-time quantitative polymerase chain reaction (RT-qPCR)

Gene amplification and identification were duplicated using a Rotorgene 6000 real-time PCR machine (Corbett, Sydney, Australia) and a Bioline SYBR Green kit Sensi FASTTM SYBR No-ROX (Bioline, Sydney, Australia). Each primer was 400 mM long, and 5 µL of 2 × Sensi FAST SYBR No-ROX was used in a 10 µL PCR reaction. Gene expression stability was assessed using the geNorm M module of qbase+ version 3.0 (Biogazelle, Zwijnbeke, Belgium), which evaluated eight housekeeping genes*: β-actin, glyceraldehyde 3-phosphate dehydrogenase* (***GAPDH***)*, hypoxanthine-guanine phosphoribosyltransferase* (***HPRT***)*, hydroxymethylbilane synthase* (***HMBS***)*, TATA box-binding protein* (***TBP***)*, tyrosine 3-monooxygenase/tryptophan 5-monooxygenase* (***YWHAZ***)*, Succinate Dehydrogenase Subunit A* (***SDHA***)*, Ribosomal protein L4* (***RPL4***)*,* and *β-actin* (***ACTB***). *GAPDH* (M-value = 0.125) and *SDHA* (M-value = 0.145) were identified as the two most stable reference genes for normalizing target gene expression. Amplification cycle (Cq) values for target genes were collected and analyzed with qbase+ software (Biogazelle, Zwijnbeke, Belgium), which uses the arithmetic mean method to convert logarithmic Cq values to linear relative quantities using the exponential function for gene quantification (Hellemans et al., 2007; Vandesompele et al., 2002). Normalized relative quantities (NRQ) for each target gene were calculated and analyzed across all samples. The primers used in this study are listed in supplementary data Table S2.

### Caecal DNA isolation

DNA was extracted from frozen caecal samples collected on d16 using the QIAamp DNA Stool kit (Qiagen, Hilden, Germany) following the manufacturer’s protocol. Briefly, 80 mg of caecal digesta and 300 mg of BioSpec glass beads (0.1 mm) (Daintree Scientific, St Helens, Tasmania, Australia) were placed into 2 mL microcentrifuge tubes. Each sample was resuspended in 500 μL of pre-warmed buffer ASL and subjected to bead beating using a Tissuelyser II (Qiagen, Hilden, Germany) for 4 min at 30 Hz. Samples were then incubated at 90°C for 10 minutes, vortexed for 15 s, and centrifuged at 20,000 × *g* for 1 min. The supernatant was transferred to a new 2 mL microcentrifuge tube, mixed with 300 μL of InhibitEx solution, vortexed for 1 min, and incubated at room temperature for 1 minute to allow inhibitors to adsorb to the InhibitEx matrix. The supernatant was transferred to a fresh 2 mL tube and centrifuged at 20,000 × *g* for 3 min. Following centrifugation, 600 μL of the supernatant was transferred to a new 2 mL tube containing 30 μL of Proteinase K, and 600 μL of buffer AL was added, vortexed for 15 s, and incubated at 70°C for 10 min. The lysate was then mixed with 500 μL of 96-100% ethanol, vortexed, and centrifuged for 30 seconds before being applied to a QIAamp spin column. The column was washed with 500 μL each of buffers AW1 and AW2. After washing, the C6 buffer was used, and the column was incubated at room temperature for 2 min, followed by centrifugation at 20,000 × *g* for 1 min. DNA quantity and quality were assessed using a Nanodrop 8000 spectrophotometer (Thermo Fisher Scientific, Waltham, MA, USA). DNA with high purity (A260/A280 > 1.8) was stored at −20°C until use.

### Next-generation sequencing and microbiota data analysis

The V3-V4 region of the 16S rRNA genes was amplified using primers 341F and 805R and sequenced on the Illumina MiSeq platform with 2 × 300 bp paired-end configuration. Raw sequence quality was assessed with FastQC v0.11.9 (Babraham Institute, Cambridge, UK) (Andrews, 2010) and MultiQC v1.11 (Ewels et al., 2016). The sequencing primers were removed with the cutadapt (Martin, 2011). The processing and denoising of the sequencing data were carried out in QIIME2 v2020.6.0 (Bolyen et al., 2019) using the Divisive Amplicon Denoising Algorithm 2 (DADA2) plugin, which performs quality filtering, denoising, and removing chimeric sequences (Callahan et al., 2016). This plugin implements a robust algorithm that leverages error profiles within amplicon data to identify and remove sequencing errors, resulting in high-quality amplicon sequence variants (ASVs) for downstream analysis. The denoised ASVs were assigned a taxonomic classification based on the SILVA v138 database (Quast et al., 2013).

Downstream ecological and statistical analyses, including diversity metrics and visualization, were conducted in R v4.0.3 (R Core Team, 2013) using the packages Phyloseq (McMurdie and Holmes, 2013), Vegan (Dixon, 2003), and Microeco (Liu et al., 2021a).

### Data analysis

All data were tested for normality before statistical analysis. Analyses were performed using JMP 16.0 (SAS Institute, Cary, NC, USA). A completely randomized design with a 2 × 2 factorial arrangement was applied, and treatment means were compared using Tukey's test for significance. When the additional treatment NC was included, a one-way ANOVA analysis was used to assess the treatment effects relative to non-challenge birds. For analyses of sex as a factor, a 2 × 5 factorial arrangement was used. The means were considered significantly different when the *P*-value was <0.05, and trends were reported when 0.05 < *P* < 0.10.

## Results

### Serum FITC-d concentration

Serum FITC-d results are presented in Table 3. The factorial analysis revealed no interaction between Xy and Pb on serum FITC-d levels (*P* > 0.05), and no significant main effect (*P* > 0.05). When NC was included with four other treatments in one-way ANOVA analysis, serum FITC-d concentration was significantly higher in the CC group compared to the NC (*P* < 0.05). Birds supplemented with Xy, Pb, and their combination did not differ significantly from either CC or NC (*P* > 0.05), indicating the shifts of FITC-d concentrations by the treatments of Pb, Xy, and their combination.

**Table 3:** Effect of xylanase and *B. subtilis* on d16 serum FITC-d concentration in broilers challenged with necrotic enteritis.

1Treatments	Xylanase (%)	*B. subtilis (*%)	2FITC-d (*u*g/mL serum)
CC	No	No	0.166^a^
Pb	No	Yes	0.131^ab^
Xy	Yes	No	0.151^ab^
Xy+Pb	Yes	Yes	0.146^ab^
NC			0.117^b^
3SEM			0.011
***P-value***			0.024
**Main effects (2 × 2)**			
Xylanase (Xy)			
No			0.148
Yes			0.149
SEM			0.01
*B. subtilis* (Pb)			
No			0.158
Yes			0.139
SEM			0.01
***P*-value**			
Xy			0.975
Pb			0.108
Xy × Pb			0.208

a-b values within a column with no common superscripts differ significantly (*P* < 0.05).

1 Treatment abbreviations: CC, challenged control; Xy, challenged control+ xylanase (0.03%); Pb, challenged control+ *B. subtilis* (0.05%); Xy + Pb, challenged control+ xylanase (0.03%) + *B. subtilis* (0.05%); NC, non-challenged control.

2 FITC-d = fluorescein isothiocyanate dextran.

3 SEM: standard error of mean.

### Caecal SCFA

Table 4 presents caecal SCFA concentrations. In the factorial analysis, no significant interaction between Xy and Pb was observed for caecal SCFA concentrations (*P* > 0.05). A tendency for Xy was observed on propionate (*P* = 0.064), iso-butyrate (*P* = 0.057), and iso-valeric acid (*P* = 0.063) concentrations, where Xy supplementation tended to reduce these SCFA compared to the treatments without Xy inclusion.

**Table 4:** Effect of the xylanase and *B. subtilis* on d16 caecal SCFA in broilers challenged with necrotic enteritis.

1Treatments	Xylanase (Xy) %	*B. subtilis* (Pb) %	Formate	Acetate	Propionate	Iso-butyrate	Butyrate	Iso-valeric	Valeric	Lactate	Succinate	Total 2SCFA
CC	No	No	0.579	48.7	3.96	0.978	13.7	0.442	0.921	0.118	8.05	77.5
Pb	No	Yes	0.603	51.3	3.80	0.912	14.2	0.393	1.00	0.415	4.18	76.9
Xy	Yes	No	0.700	47.0	2.84	0.622	12.7	0.261	0.598	0.277	7.09	72.1
Xy+Pb	Yes	Yes	0.638	52.6	2.91	0.852	15.0	0.312	0.857	0.202	6.78	80.1
NC			1.33	65.7	3.60	0.573	13.7	0.166	0.727	0.226	5.96	92.0
3SEM			0.188	7.29	0.533	0.112	2.94	0.069	0.168	0.125	2.93	11.54
***P*-value**			0.070	0.485	0.424	0.064	0.987	0.080	0.449	0.523	0.902	0.832
**Main effect (2 × 2)**												
Xylanase (Xy)												
No			0.591	50.0	3.88	0.945	14.0	0.418	0.962	0.267	6.12	77.1
Yes			0.669	49.8	2.87	0.737	13.8	0.286	0.728	0.240	6.93	76.1
SEM			0.135	5.24	0.369	0.074	2.13	0.048	0.119	0.087	2.16	8.39
*B. subtilis* (Pb)												
No			0.639	47.9	3.40	0.800	13.2	0.351	0.759	0.197	7.57	74.8
Yes			0.620	51.9	3.35	0.882	14.8	0.353	0.930	0.309	5.48	78.4
SEM			0.135	5.24	0.369	0.074	2.13	0.048	0.119	0.087	2.16	8.39
***P*-value**												
Xy			0.685	0.984	0.064	0.057	0.966	0.063	0.175	0.829	0.791	0.934
Pb			0.922	0.586	0.929	0.439	0.653	0.987	0.320	0.375	0.499	0.760
Xy × Pb			0.825	0.841	0.818	0.170	0.769	0.468	0.605	0.142	0.565	0.717

^a-b^ values within a column with no common superscripts differ significantly (*P* < 0.05).

1 Treatment abbreviations: CC, challenged control; Xy, challenged control+ xylanase (0.03%); Pb, challenged control+ *B. subtilis* (0.05%); Xy + Pb, challenged control+ xylanase (0.03%) + *B. subtilis* (0.05%); NC, non-challenged control.

2 SCFA: short-chain fatty acids.

3 SEM: standard error of mean.

In the one-way ANOVA analysis, compared with the NC group, formate concentration tended to be lower in all challenged groups (*P* = 0.070). However, iso-butyrate (0.064) and iso-valerate (0.080) concentrations tended to increase in all NE-challenged groups compared to the NC.

### Ileal SCFA

Ileal SCFA concentrations are presented in Table 5. Factorial analysis revealed a tendency for an interaction between Xy and Pb (*P* = 0.053) for succinate concentration, while no other significant interaction was observed. Dietary Pb supplementation showed a significant main effect on lactate (*P* < 0.05) and total SCFA (*P* < 0.05) concentrations. Supplementation of Pb significantly increased lactate (*P* < 0.05) and total SCFA (*P* < 0.05) concentrations compared to the treatments without its supplementation. Moreover, Pb showed a tendency to affect ileal butyrate concentration (*P* = 0.082), whereas supplementation of Pb tended to reduce the concentration compared to the treatment groups without its supplementation.

**Table 5:** Effect of the xylanase and *B. subtilis* on d16 ileal SCFA concentrations in broilers challenged with necrotic enteritis.

1Treatments	Xylanase (Xy) %	*B. subtilis* (Pb) %	Formate	Acetate	Propionate	Iso-butryrate	Butyrate	Lactate	Succinate	Total 2SCFA
CC	No	No	0.600	2.71	0.236	0.002	0.048	34.9^a^	0.363^a^	38.8^a^
Pb	No	Yes	0.587	2.83	0.234	0.000	0.027	42.1^a^	0.315^ab^	46.1^a^
Xy	Yes	No	0.950	2.29	0.201	0.172	0.047	23.3^ab^	0.234^ab^	27.2^ab^
Xy+Pb	Yes	Yes	0.632	3.16	0.243	0.000	0.004	39.5^a^	0.404^a^	43.9^a^
NC			0.475	2.39	0.256	0.000	0.003	5.88^b^	0.098^b^	9.10^b^
3SEM			0.249	0.370	0.026	0.081	0.016	4.91	0.054	5.10
***P*-value**			0.719	0.470	0.666	0.455	0.148	<0.0001	0.003	<0.0001
**Main effect (2 × 2)**										
Xylanase (Xy)										
No			0.593	2.77	0.235	0.001	0.038	38.5	0.339	42.5
Yes			0.791	2.72	0.222	0.086	0.025	31.4	0.319	35.6
SEM			0.193	0.285	0.018	0.063	0.013	3.79	0.038	3.94
*B. subtilis* (Pb)										
No			0.775	2.50	0.219	0.087	0.047	29.1^b^	0.298	33.0^b^
Yes			0.610	2.99	0.238	0.000	0.016	40.8^a^	0.360	45.0^a^
SEM			0.193	0.285	0.018	0.063	0.013	3.79	0.038	3.94
***P*-value**										
Xy			0.473	0.917	0.623	0.346	0.498	0.195	0.714	0.225
Pb			0.549	0.235	0.450	0.334	0.082	0.037	0.268	0.039
Xy × Pb			0.580	0.364	0.401	0.346	0.564	0.410	0.053	0.402

a-b values within a column with no common superscripts differ significantly (*P* < 0.05).

1 Treatment abbreviations: CC, challenged control; Xy, challenged control+ xylanase (0.03%); Pb, challenged control+ *B. subtilis* (0.05%); Xy + Pb, challenged control+ xylanase (0.03%) + *B. subtilis* (0.05%); NC, non-challenged control.

2 SCFA: short-chain fatty acids.

3 SEM: standard error of mean.

When comparing NC with four other treatments in one-way ANOVA analysis, ileal lactate (*P* < 0.05) and total SCFA (*P* < 0.05) were significantly higher in the CC, Pb, and Xy+Pb groups than in the NC group (*P* < 0.05). Succinate concentration was significantly higher in the CC and Xy+Pb groups compared with NC (*P* < 0.05).

### Jejunal gene expression

The expression of genes related to tight junction proteins, immunity, apoptosis, and mucin protein is presented in Table 6. The 2 × 2 factorial analysis revealed no significant Xy × Pb interactions (*P* > 0.05) or main effects (*P* > 0.05) on any of the genes (Table 6). When the NC treatment was included for one-way ANOVA analysis with the other treatments, *IgA* expression of Pb, Xy, and Xy+Pb was decreased compared to the NC group (*P* < 0.05). The *MUC2* gene was downregulated by the treatments of Pb, Xy, Xy+Pb, and CC compared to the NC group (*P* < 0.05).

**Table 6:** Effect of xylanase and *B. subtilis* on d16 tight junction, immunity, apoptotic, and mucin gene expression in broilers challenged with necrotic enteritis.

1Treatments	Xylanase (Xy) %	*B. subtilis* (Pb) %	2Tight junction proteins			Cell apoptosis		Immunity			Mucin
			*TJP1*	*OCLN*	*JAM2*	*CASP3*	*CASP8*	*IgA*	*IgG*	*IgM*	*MUC2*
CC	No	No	1.38	1.35	1.05	1.23	1.16	1.49^ab^	1.56	1.05	0.995^b^
Pb	No	Yes	1.06	1.11	1.07	1.18	1.20	1.14^b^	1.70	1.35	0.938^b^
Xy	Yes	No	1.14	1.08	1.03	1.05	0.987	1.25^b^	1.20	1.04	1.08^b^
Xy + Pb	Yes	Yes	1.26	1.33	1.15	1.26	1.30	1.02^b^	1.44	1.70	1.10^b^
NC			1.48	1.06	1.35	1.18	0.865	2.30^a^	2.07	1.18	1.73^a^
3SEM			0.151	0.174	0.128	0.167	0.237	0.261	0.329	0.258	0.128
***P-*value**			0.272	0.611	0.381	0.924	0.705	0.007	0.417	0.366	<0.001
**Main effect (2 × 2)**											
Xylanase (Xy)											
No			1.22	1.23	1.06	1.20	1.18	1.33	1.70	1.22	0.967
Yes			1.23	1.21	1.10	1.17	1.13	1.16	1.35	1.35	1.09
SEM			0.136	0.134	0.096	0.134	0.181	0.199	0.277	0.202	0.097
*B. subtilis* (Pb)											
No			1.28	1.21	1.04	1.14	1.08	1.39	1.45	1.07	1.04
Yes			1.18	1.23	1.12	1.23	1.22	1.10	1.60	1.51	1.02
SEM			0.136	0.134	0.096	0.134	0.181	0.199	0.277	0.202	0.097
***P-*value**											
Xy			0.937	0.921	0.769	0.866	0.840	0.546	0.384	0.649	0.365
Pb			0.604	0.939	0.573	0.628	0.580	0.312	0.695	0.129	0.878
Xy × Pb			0.245	0.181	0.689	0.459	0.553	0.892	0.951	0.512	0.796

a-b values within a column with different letters differ significantly (*P* < 0.05).

1 Treatment abbreviations: CC, challenged control; Xy, challenged control+ xylanase (0.03%); Pb, challenged control+ *B. subtilis* (0.05%); Xy + Pb, challenged control+ xylanase (0.03%) +*B. subtilis* (0.05%); NC, non-challenged control.

2 Genes name: *TJP1 (ZO-1)*: tight junction protein 1 (Zonula occludens-1); *OCLN*: occluding; *JAM2*: junctional adhesion molecule 2; *CASP3*: Caspase-3; *CASP8*: Caspase-8; *IgA*: immunoglobulin A; *IgG*: immunoglobulin G; *IgM*: immunoglobulin M; *MUC2*: Mucin 2.

3 SEM: standard error of mean.

Table 7 presents the expression of nutrient transporter genes and inflammatory cytokines. Except for the tendency for an Xy × Pb interaction in *GLUT2* expression (*P* = 0.087) and a tendency for a main effect of Xy on *IFN-γ* (*P* = 0.080), no other significant interactions (*P* > 0.05) or main effects (*P* > 0.05) were observed for any of the genes. The expression of *IFN-γ* tended to be lower with the supplementation of Xy as the main effect compared to the treatments without its supplementation. When comparing NC with the other treatments using one-way ANOVA, the expression of *b0+AT* was significantly decreased in the Xy group (*P* < 0.05) compared with NC, while *GLUT2* expression was significantly reduced in both Pb (*P* < 0.05) and Xy (*P* < 0.05) groups.

**Table 7:** Effect of xylanase and *B. subtilis* on d16 nutrient transporter and inflammatory gene expression in the jejunum of broilers challenged with necrotic enteritis.

1Treatments	Xylanase (Xy) %	*B. subtilis* (Pb) %	2Nutrient transporter							Inflammatory	
			*ASCT1*	*bo+AT*	*LAT1*	*PepT1*	*PepT2*	*BOAT*	*GLUT2*	*IL2*	*IFN-y*
CC	No	No	1.31	1.07^ab^	1.35	1.36	0.761	0.965	1.58^ab^	1.18	1.62
Pb	No	Yes	1.43	1.06^ab^	1.11	1.91	1.42	1.19	1.20^b^	1.53	1.39
Xy	Yes	No	1.21	0.870^b^	1.30	1.51	0.732	1.03	1.15^b^	0.897	1.26
Xy + Pb	Yes	Yes	0.912	1.29^ab^	1.26	1.75	1.04	1.07	1.63^ab^	1.31	0.992
NC			0.790	1.65^a^	0.786	1.17	0.554	1.52	2.01^a^	0.691	0.812
SEM			0.275	0.167	0.146	0.503	0.271	0.159	0.192	0.393	0.241
***P-*value**			0.418	0.016	0.052	0.842	0.188	0.117	0.012	0.581	0.199
**Main effect (2 × 2)**											
Xylanase (Xy)											
No			1.37	1.06	1.23	1.59	1.07	1.08	1.39	1.41	1.49
Yes			1.08	1.09	1.29	1.63	0.879	1.05	1.51	1.11	1.13
3SEM			0.202	0.119	0.116	0.364	0.205	0.113	0.201	0.319	0.146
*B. subtilis* (Pb)											
No			1.26	0.969	1.32	1.44	0.755	1.00	1.44	1.05	1.43
Yes			1.19	1.19	1.20	1.78	1.20	1.13	1.46	1.47	1.19
SEM			0.202	0.119	0.116	0.364	0.205	0.113	0.201	0.319	0.146
***P-*value**											
Xy			0.327	0.857	0.739	0.943	0.509	0.880	0.681	0.518	0.080
Pb			0.827	0.201	0.435	0.514	0.137	0.405	0.932	0.362	0.247
Xy × Pb			0.597	0.180	0.499	0.828	0.535	0.561	0.087	0.940	0.958

a-b values within a column with different letters differ significantly (*P* < 0.05).

1 Treatment abbreviations: CC, challenged control; Xy, challenged control+ xylanase (0.03%); Pb, challenged control+ *B. subtilis* (0.05%); Xy + Pb, challenged control+ xylanase (0.03%) +*B. subtilis* (0.05%); NC, non-challenged control.

2 Gene name: *B0AT*: solute carrier family 6, member14, *ASCT1*: alanine, serine, cysteine, and threonine transporter; *bo; +AT*: solute carrier family 7, member 9; *GLUT2*: glucose transporter-2; *IFN-γ*: Interferon-gamma; *LAT1*: L type amino acid transporter-1; *PepT1*: peptide transporter-1; *PepT2*: Peptide Transporter 2.

3 SEM: standard error of mean.

**Fig. 1:**
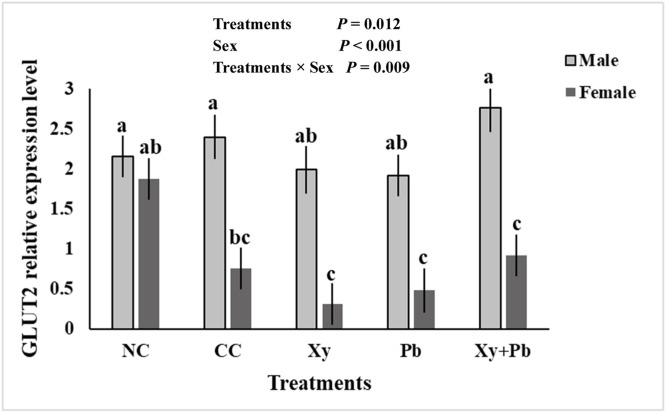
Interactions between experimental treatment and sex on *GLUT2* jejunal gene expressions. ^1^Treatments abbreviations: CC, challenged control; Xy, challenged control+ xylanase (0.03%); Pb, challenged control+ *B. subtilis* (0.05%); Xy + Pb, challenged control+ xylanase (0.03%) +*B. subtilis* (0.05%); NC, non-challenged control. *GLUT2*: glucose transporter-2. ^a-c^values within a column with different letters differ significantly (*P* < 0.05).

When sex was considered as a factor, a significant sex × treatment interaction for *GLUT2* expression was observed (*P* < 0.01) (Fig. 1). While *GLUT2* expression levels were similar between sexes in the NC group (*P* > 0.05), all NE-challenged females exhibited significantly reduced *GLUT2* expression compared to males (*P* < 0.05).

### Caecal microbiota

**Fig. 2:**
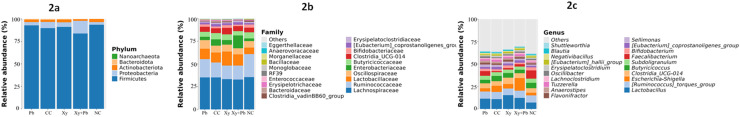
**(a-c)**: Bacterial distribution in d16 caecal content across different treatments at different taxonomic levels (a, at phylum level; b, at family level; c, at genus level).CC, challenged control; Xy, challenged control+ xylanase (0.03%); Pb, challenged control+ *B. subtilis* (0.05%); Xy + Pb, challenged control+ xylanase (0.03%) +*B. subtilis* (0.05%); NC, non-challenged control.

A total of 90 caecal DNA samples from five treatment groups (45 males and 45 females) were sequenced to investigate differences in the caecal microbiota composition. At the phylum level (Fig. 2a and supplementary data Table S3), the four most abundant phyla were Firmicutes, Proteobacteria, Actinobacteria, and Bacteroidota, with Firmicutes being the most abundant phylum across all treatments. Firmicutes tended to differ among treatment groups (*P* = 0.082), with the highest levels observed in NC group (93.7%), followed closely by Xy and Pb (91.0% each), CC (89.9%), and Xy+Pb (85%). Proteobacteria ranked second and showed a significant difference (*P* < 0.05) among treatments, with the highest abundance in the Xy+Pb group (12.3%) compared to NC (2.85%). However, the relative abundance in Xy+Pb was not significantly different (*P* > 0.05) from that in CC (7.1%), Pb (5.6%), and Xy (5.3%) groups. Actinobacteria ranked third and did not differ significantly between treatments (*P* > 0.05). Bacteroidota was found in all NE-challenged groups at less than 1%, but was not found at the detectable level in the NC group, and this difference was not significant (*P* > 0.05).

At the family level (Fig. 2b and supplementary data Table S4), Lachnospiraceae was the dominant family across all treatments, however, with no significant differences among groups (*P* > 0.05). Ruminococcaceae was the second most abundant family, showing a significantly higher abundance (*P* < 0.05) in the NC group (24.7%) compared to CC (16.9%) group. Pb-supplemented birds showed an intermediate level (19.3%), which did not differ significantly from either NC or CC groups. Lactobacillaceae ranked third, with no significant differences (*P* > 0.05) between NC (7.7%) and CC (12.2%) birds. However, the birds supplemented with Xy (21.8%) group had significantly higher abundance (*P* < 0.05) of Lactobacillaceae compared to NC (7.7%) group. Oscillospiraceae showed consistent proportions (*P* > 0.05) across treatments, ranging from 6% to 9%. In Contrast, Enterobacteriaceae was significantly more abundant (*P* < 0.05) in the Xy+Pb group (12.2%) than the NC group (2.81%), but did not differ from the CC, Xy, and Pb groups (*P* > 0.05).

At the genus level (Fig. 2c and supplementary data Table S5), 92 genera were identified. Among the top 20 genera, *Lactobacillus* was the most abundant, with significantly higher (*P* < 0.05) relative abundance in the Xy group (21.8%) compared to NC (7.7%), but not different from the CC (12.2%) group. The *Ruminococcus torques* group ranked second, ranging from 6% to 8%, showing similar abundances across treatments (*P* > 0.05). *Escherichia-Shigella* was the third most abundant genus and showed no difference between CC and NC groups, but with a significantly higher abundance (*P* < 0.05) in the Xy+Pb (12.2%) compared to the NC group (2.8%). *Fecalibacterium* abundance was significantly higher (*P* < 0.05) in the NC group (8.8%) compared to the CC (1.3%). Interestingly, Pb and Xy+Pb supplementations shifted *Fecalibacterium* abundance (3.8%) towards NC levels, showing no significant difference from NC and CC groups (*P* > 0.05).

**Fig. 3:**
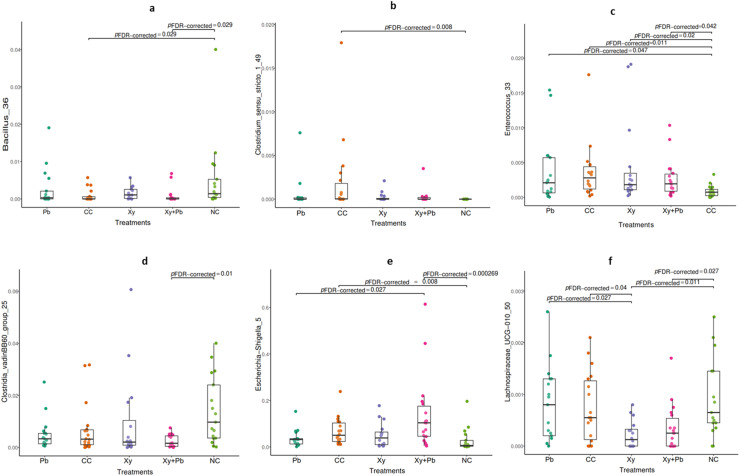
(a-f): Univariate analysis of the effect of xylanase and *B. subtilis* supplementation on d16 caecal bacteria composition (family and genus level) in broilers challenged with necrotic enteritis. CC, challenged control; Xy, challenged control+ xylanase (0.03%); Pb, challenged control+ *B. subtilis* (0.05%); Xy + Pb, challenged control+ xylanase (0.03%) +*B. subtilis* (0.05%); NC, non-challenged control.

Univariate pairwise comparisons of taxa using the Dunn’s test revealed significant (*P* < 0.05) treatment effects at both the family and genus levels (Fig. 3a-f). The NE challenge significantly increased the abundance of *Enterococcus* (*P* < 0.05), *Clostridium sensu stricto* 1 (*P* < 0.05), and *Escherichia-Shigella* (*P <* 0.05), while significantly decreasing *Bacillus* (*P* < 0.05) in the CC group compared to NC group. Birds fed with Xy alone had reduced *Lacnospiraceae* UCG 010 (*P <* 0.05) compared to the CC group (*P* < 0.05).

### Alpha and beta diversity

**Fig. 4:**
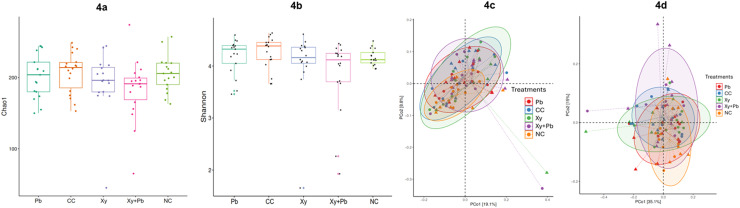
**(a-d)**: Effects of xylanase and *B. subtilis* on d16 caecal microbial diversity: (a) Chao1, (b) Shannon, (c) unweighted UniFrac, and (d) weighted UniFrac principal coordinates analysis (PCoA) plots. CC, challenged control; Xy, challenged control+ xylanase (0.03%); Pb, challenged control+ *B. subtilis* (0.05%); Xy + Pb, challenged control+ xylanase (0.03%) +*B. subtilis* (0.05%); NC, non-challenged control.

As shown in Fig. 4a and 4b, caecal microbiota richness and diversity were assessed using the Chao 1 and Shannon indices, and no significant treatment effects were observed (*P* > 0.05). Beta diversity analysis was performed to assess the differences in microbial communities among the five treatment groups. Principal coordinates analysis (PCoA) based on both unweighted and weighted UniFrac metrics revealed no clear visual separation among groups (Fig. 4c and 4d). However, pairwise permutational multivariate analysis of variance (**PERMANOVA**) based on both unweighted and weighted UniFrac metrices showed a significant difference between the NC and CC groups (*P* < 0.05) (Table 8). Weighted UniFrac analysis showed that microbial communities in NC differed significantly from those in the CC, Xy, and Xy+Pb groups (*P* < 0.05). Similarly, unweighted UniFrac analysis also demonstrated significant differences between NC and several challenged groups, including CC, Pb, and Xy+Pb (*P* < 0.05). In contrast, no significant differences were detected among the challenged treatment groups under either metric (*P* > 0.05), suggesting that while NE challenge markedly altered caecal microbiota composition compared with the non-challenged control, supplementation with Xy, Pb, or their combination did not fully restore the microbiota structure to that of healthy birds.

**Table 8:** PERMANOVA-based comparison of microbial community structure between groups using multiple distance metrics.

1Treatment groups	Unweighted- UniFrac		Weighted- UniFrac	
	R^2^	Adjusted-P	R^2^	Adjusted-P
NC vs Xy	0.052	0.100	0.086	0.027
NC vs Xy+Pb	0.069	0.030	0.105	0.015
NC vs CC	0.060	0.040	0.114	0.015
NC vs Pb	0.054	0.040	0.053	0.230
Xy vs Xy+Pb	0.026	0.749	0.029	0.610
Xy vs CC	0.031	0.684	0.021	0.629
Xy vs Pb	0.025	0.749	0.023	0.629
Xy+Pb vs CC	0.027	0.684	0.026	0.629
Xy+Pb vs Pb	0.028	0.684	0.050	0.230
CC vs Pb	0.023	0.749	0.037	0.443

PERMANOVA= Permutational multivariate analysis of variance; R^2^ is the proportion of the variance explained by the group.

1 Treatment abbreviations: CC, challenged control; Xy, challenged control+ xylanase (0.03%); Pb, challenged control+ *B. subtilis* (0.05%); Xy + Pb, challenged control+ xylanase (0.03%) +*B. subtilis* (0.05%); NC, non-challenged control.

**Fig. 5:**
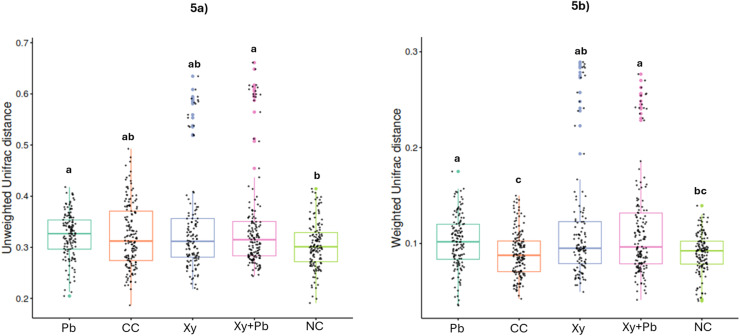
**(a-b)**: Effect of xylanase and *B. subtilis* supplementation on beta diversity (weighted and unweighted UniFrac distance matrices) in d16 broilers challenged with necrotic enteritis. CC, challenged control; Xy, challenged control+ xylanase (0.03%); Pb, challenged control+ *B. subtilis* (0.05%); Xy + Pb, challenged control+ xylanase (0.03%) +*B. subtilis* (0.05%); NC, non-challenged control. ^a-c^values within a column with different letters differ significantly (*P* < 0.05).

Within-group (sample-to-sample) distance analysis based on both unweighted and weighted UniFrac metrics revealed significant differences in microbial community dispersion among treatment groups. For the unweighted UniFrac distances, significant differences were detected between the Pb and NC groups, and between the Xy+Pb and NC groups, indicating that both treatments increased within-group variability compared with the non-challenged control (Fig. 5a). This suggests greater individual variation in microbial community composition following NE challenge and partial modulation by Pb or the combined supplementation. For the weighted UniFrac distances, significant differences were observed between the Pb, Xy+Pb, and Xy groups compared with the CC group, as well as between the Pb and NC groups and between the Xy+Pb and NC groups (Fig. 5b). These findings indicate that supplementation, particularly with Pb or the combination of Xy and Pb, altered microbial dispersion relative to both the challenged and non-challenged controls.

## Discussion

Necrotic enteritis (NE) remains a major challenge to poultry health and productivity in the post-antibiotic era, highlighting the need for effective alternative strategies to maintain gut integrity and mitigate disease impacts. This study evaluated the individual and combined effects of xylanase (Xy) and probiotic (Pb) supplementation in alleviating NE-associated gut damage in broilers. The current study builds on our previous study (Khairunnesa et al., 2025), which confirmed a successful induction of subclinical NE, as evidenced by reduced body weight gain, increased feed conversion ratio, and elevated intestinal lesions and impaired gut histomorphology in challenged birds (CC) relative to the non-challenged control (NC).

Consistent with NE pathology, the present findings confirmed classical signs of intestinal barrier dysfunction, including elevated serum FITC-d levels, downregulation of mucosal defence genes (*MUC2* and *IgA*), shifts in caecal microbial composition, and altered SCFA profiles and dysbiosis, which are key features of NE infection (Akerele et al., 2022; Gharib-Naseri et al., 2021; Kumar et al., 2022). Both Xy and Pb supplementation showed improvements in gut health markers, but Pb exhibited more consistent and significant effects across microbial diversity and SCFA production. In contrast, the combined supplementation did not exhibit synergistic benefits, suggesting overlapping or competing mechanisms. Overall, these results partially support the hypothesis that Xy and Pb individually promote beneficial modulation of gut microbiota and mitigate NE-associated intestinal damage, but do not support synergistic effects when both are combined.

Pb supplementation enhanced gut health under NE challenge, evidenced by notable changes in microbiota composition, increased ileal lactate and total SCFA, and partial restoration of intestinal integrity. UniFrac analyses revealed a distinct separation of the Pb group from CC, suggesting a shift in microbial community structure. This was evidenced by a greater abundance of health-promoting bacterial groups such as Ruminococcaceae and *Fecalibacterium*, and a 21.1% (CC, 7.1% vs Pb 5.6%) relative reduction in Proteobacteria compared to CC, a phylum commonly associated with dysbiosis and inflammation (Shin et al., 2015). These microbial shifts were accompanied by elevated ileal lactate and total SCFA concentrations, indicating increased fermentative microbial activity, consistent with earlier reports on Pb (Guo et al., 2020; Qiu et al., 2021; Wang et al., 2021c). However, unlike Aljumaah et al. (2020), who reported higher caecal acetate and butyrate levels, such increases were not evident in the present study, possibly reflecting differences in gut region and the Pb’s dose provided to the birds. The current increase in ileal lactate and total SCFA production is likely due to the influence of Pb on *Lactobacillus* and other fermentative taxa, such as Ruminococcaceae and *Fecalibacterium,* as *Bacillus* species are known to promote the growth of such beneficial bacteria (Grant et al., 2018; Knarreborg et al., 2008). Moreover, Pb supplementation shifted FITC-d levels towards those of NC birds, indicating partial restoration of gut permeability and mucosal integrity. Collectively, these microbial shifts towards a more beneficial microbial profile, along with increased SCFA production and reduced intestinal permeability, provide strong evidence that Pb helped restore gut balance under NE challenge. Therefore, these improvements account for the improved performance observed in Pb-supplemented broilers (Khairunnesa et al., 2025).

Dietary supplementation with Xy also modulated microbial dysbiosis, reducing 25.4% Proteobacteria, a phylum associated with pathogenic bacteria and inflammation (Shin et al., 2015). Additionally, it showed a tendency to reduce branched-chain fatty acids and the expression of *IFN-γ*, and significantly increased Lachnospiraceae UCG 010. The relative abundance of Proteobacteria decreased from 7.1% in the CC group to 5.3% in the Xy-supplemented group, corresponding to a 25.4% reduction. This difference suggests a shift towards improved microbial balance, consistent with the results of Wang et al. (2021b). Considering that Proteobacteria are often associated with gut dysbiosis and inflammation, their reduction may partially explain the tendency to reduce *IFN-γ* expression with Xy supplementation, indicating a possible anti-inflammatory effect. Although not statistically confirmed, this finding complements the microbiota findings and may reflect a mild immunomodulatory role for Xy in NE-challenged birds. Furthermore, Xy tended to reduce caecal branched-chain fatty acids (**BCFAs**) levels, such as iso-butyrate and iso-valerate, which are derived from microbial fermentation of branched-chain amino acids in the hindgut (Ríos-Covián et al., 2016; Smith and Macfarlane, 1997). Elevated BCFAs indicate excessive protein fermentation in the hindgut, leading to the production of nitrogenous metabolites that may impair gut integrity (Russell et al., 2011). In this study, however, the reduction tendency in BCFAs alone was insufficient to completely alleviate the compromised gut barrier associated with the NE challenge, but it was partially restored, as indicated by the shifting FITC-d. Despite these mentioned modulations, Xy did not significantly increase SCFA levels in either the ceca or the ileum, possibly due to rapid absorption of SCFA in the ileum, which limits their availability for fermentation in the caeca (Den Besten et al., 2013; González-Ortiz et al., 2019). Notably, Xy supplementation significantly reduced the abundance of Lachnospiraceae UCG 010, a bacterial family known to contribute to SCFA production and performance improvement (Lau et al., 2024; Liu et al., 2021b; Popov et al., 2024). Members of the Lachnospiraceae degrade plant-derived cellulose and hemicellulose into acetate, butyrate, and propionate, which are key energy sources that support gut health and nutrient utilization (Biddle et al., 2013). Interestingly, this result contrasts with findings in pigs, where Xy supplementation increased Lachnospiraceae abundance in high-fibre diets (Petry et al., 2021). This discrepancy may be due to species-specific responses, fibre source, and level of use, and disease state (i.e., NE challenge). The reduction in *Lachnospiraceae* UCG 010 may have contributed to the lower SCFA concentrations and limited nutrient absorption, which could help explain the absence of significant performance improvements with Xy (Khairunnesa et al., 2025) despite some microbiota modulations and indications of improved gut health.

Consistent with the performance results of this study (Khairunnesa et al., 2025), no synergistic effects of Xy and Pb supplementation on FITC-d levels, SCFA concentrations, and microbial diversity were observed. Similarly, Machado et al. (2020) reported no associative effect of xylanase and probiotics on microbiota modulation. While Xy breaks down xylans into fermentable sugars, Pb may have limited access to these sugars in the caeca for optimal fermentation. This could be due to competition for substrates between Pb and other microbes, along with the rapid absorption of short-chain fatty acids produced by Xy in the upper intestine, which likely limits Pb’s ability to utilize the sugars effectively. Additionally, overlapping microbial modulation pathways could saturate the microbiota's ability to respond, reducing the expected combined benefit. These findings highlight the importance of understanding the interactions between dietary additives to optimize their combined efficacy.

Another interesting finding was the significant treatment × sex interaction for a glucose transporter gene, *GLUT2*, with female birds in all NE-challenged groups exhibiting significantly lower expression than males. Kaminski and Wong (2017) previously found that male chickens had higher *GLUT2* expression in the jejunum on d7, with no sex difference on d14 under normal conditions. The higher expression of this gene in males may reflect their generally faster growth rates and greater demand for nutrient uptake (England et al., 2023). However, females are known to have a more efficient immune response than males (Leitner et al., 1989; Maggini et al., 2018), which could influence metabolic priorities during the NE challenge. Under inflammatory conditions such as NE, females may divert metabolic energy toward immune functions, such as cytokine production and tissue repair, rather than nutrient absorption. This immunometabolic reprogramming could explain the downregulation of *GLUT2*, in females, as glucose transport may be deprioritized in favour of maintaining immune defence.

## Conclusions

In conclusion, Pb supplementation improved gut health in NE-challenged broilers by increasing total SCFA and lactate concentrations, promoting beneficial microbiota, and showing shifts in enhancing intestinal barrier function. Xy supplementation modulated the gut microbiota profile and showed trends toward reduced BCFAs and improved gut integrity. The absence of a synergistic effect with combined Pb and Xy may suggest overlapping mechanisms of microbial modulation. Future research should focus on optimizing the dosage and combination strategies of Xy alone and Pb to maximize their benefits on gut health and productivity under NE challenge.

## CRediT authorship contribution statement

**Most Khairunnesa:** Writing – original draft, Software, Methodology, Formal analysis, Data curation. **Alip Kumar:** Writing – review & editing, Supervision, Methodology, Investigation, Data curation. **Shu-Biao Wu:** Writing – review & editing, Supervision, Investigation, Data curation. **Mingan Choct:** Writing – review & editing, Supervision, Data curation. **Yadav Sharma Bajagai:** Writing – review & editing, Methodology, Data curation. **Kosar Gharib-Naseri:** Writing – review & editing, Visualization, Supervision, Methodology, Data curation.

## Disclosures

We declare that we have no financial and personal relationships with other people or organizations that can inappropriately influence our work, there is no professional or other personal interest of any nature or kind in any product, service and/or company that could be construed as influencing the content of this paper.

## Appendix Supplementary materials

Image, application 1

Image, application 2

Image, application 3

Image, application 4

Image, application 5
