# Supplementary material for: Xylanase and Bacillus subtilis PB6 modulate microbiota and short-chain fatty acid profiles in broilers under necrotic enteritis-challenge
Source: Poult Sci. 2025 Dec 22;105(2):106330. doi: 10.1016/j.psj.2025.106330 (PMC12805170; doi:10.1016/j.psj.2025.106330)
Supplement: Supplementary file 1 [file mmc1.docx]

| Analyzed nutrients | ^1^Treatments | | | | |
| --- | --- | --- | --- | --- | --- |
|  | NC CC Xy Pb Xy+Pb | | | | |
| Gross energy (MJ/Kg DM) |  |  |  |  |  |
| Starter | 18.9 | 18.9 | 18.9 | 18.8 | 18.7 |
| Grower | 18.7 | 18.7 | 18.8 | 18.9 | 18.8 |
| Finisher | 19.1 | 19.1 | 18.8 | 19.1 | 18.9 |
| Crude protein %, DM basis |  |  |  |  |  |
| Starter | 24.8 | 24.8 | 25.5 | 25.9 | 24.9 |
| Grower | 23.3 | 23.3 | 23.9 | 23.9 | 23.9 |
| Finisher | 21.5 | 21.5 | 22.1 | 22.0 | 21.5 |

**Supplementary Table S1**: Analyzed nutrients of starter (d0-8), grower (d9-19) and finisher (d20-35) feed

DM=dry matter; ^1^Treatment abbreviations: NC= non-challenge; Xy, challenged control+ xylanase (0.03%); Pb, challenged control+ *B*. *subtilis* (0.05%); Xy + Pb, challenged control+ xylanase (0.03%) +*B*. *subtilis* (0.05%); NC, non-challenged control.
