# Supplementary material for: Xylanase and Bacillus subtilis PB6 modulate microbiota and short-chain fatty acid profiles in broilers under necrotic enteritis-challenge
Source: Poult Sci. 2025 Dec 22;105(2):106330. doi: 10.1016/j.psj.2025.106330 (PMC12805170; doi:10.1016/j.psj.2025.106330)
Supplement: Supplementary file 2 [file mmc2.docx]

**Supplementary Table S2**: Primer’s sequence used for quantitative real-time PCR

| Genes | Sequence (5'→3') | Amplicon Size  (bp) | Ta  (^0^C) | | Reference |
| --- | --- | --- | --- | --- | --- |
| Target genes | | |  |  | |
| *^1^TJP1* | F-GGATGTTTATTTGGGCGGC  R-GTCACCGTGTGTTGTTCCCAT | 187 | 60 | (Zanu et al., 2020) | |
| *OCLN* | F-ACGGCAGCACCTACCTCAA  R-GGGCGAAGAAGCAGATGAG | 123 | 60 | (Du et al., 2016) | |
| *JAM2* | F-AGACAGGAACAGGCAGTGCTAG  R-ATCCAATCCCATTTGAGGCTAC | 135 | 60 | (Kumar et al., 2021) | |
| *CASP3* | F-TGGTGGAGGTGGAGGAGC  R- GTTTCTCTGTATCTTGAAGCACCA | 110 | 62 | (Gharib-Naseri et al., 2020) | |
| *CASP8* | F- GGAGCTGCTCTATCGGATCAAT  R- AGCAGATACCTGAACGGAGACAC | 126 | 60 | (Gharib-Naseri et al., 2020) | |
| *IgA* | F- GTCACCGTCACCTGGACACCA  R- ACCGATGGTCTCCTTCACATC | 192 | 64 | (Lammers et al., 2010) | |
| *IgG* | F- ATCACGTCAAGGGATGCCCG  R- ACCAGGCACCTCAGTTTGG | 118 | 60 | (Zhao et al., 2013) | |
| *IgM* | F- GCATCAGCGTCACCGAAAGC  R- TCCGCACTCCATCCTCTTGC | 98 | 60 | (Zhao et al., 2013) | |
| *MUC2* | F- CCCTGGAAGTAGAGGTGACTG  R- TGACAAGCCATTGAAGGACA | 143 | 60 | (Fan et al., 2015) | |
| *B^0^AT* | F-GTGTTTGGAACCCTAAATACGAGG  R- TAGCATAGACCCAGCCAGGA | 72 | 60 | (Kheravii et al., 2018) | |
| *GLUT2* | F- TGATCGTGGCACTGATGGTT  R- CCACCAGGAAGACGGAGATA | 171 | 60 | (Kheravii et al., 2018) | |
| *IFN-γ* | F-AGCTGACGGTGGTGGACCTATTATT  R- GGCTTTGCGCTGGATTC | 259 | 60 | (Li et al., 2018) | |
| *ASCT1* | F-TTGGCCGGGAAGGAGAAG  R-AGACCATAGTTGCCTCATTGAATG | 63 | 60 | (Paris and Wong, 2013) | |
| *b^0^****^,^****+AT* | F-CAGTAGTGAATTCTCTGAGTGTGAAGCT  R-GCAATGATTGCCACAACTACCA | 88 | 60 | (Gilbert et al., 2007) | |
| *LAT1* | F-GATTGCAACGGGTGATGTGA  R-CCCCACACCCACTTTTGTTT | 70 | 60 | (Gilbert et al., 2007) | |
| *PepT1* | F-TACGCATACTGTCACCATCA  R-TCCTGAGAACGGACTGTAAT | 205 | 60 | (Guo et al., 2014) | |
| *PepT2* | F-TGACTGGGCATCGGAACAA  R-ACCCGTGTCACCATTTTAACCT | 63 | 60 | (Paris and Wong, 2013) | |
| Reference genes | | |  |  | |
| *GAPDH* | F-GAAGCTTACTGGAATGGCTTTCC  R-CGGCAGGTCAGGTCAACAA | 66 | 61 | (Kuchipudi et al., 2012) | |
| *SDHA* | F-ATACGGGAAGGAAGGGGTTG  R-TGCTGGGGTGGTAAATGGTG | 74 | 60 | (Barzegar et al., 2021) | |

^1^Genes name: *TJP1 (ZO-1)*: tight junction protein 1 (Zonula occludens-1); *OCLN*: occluding; *JAM2*: junctional adhesion molecule 2; *CASP3*: Caspase-3; *CASP8*: Caspase-8; *IgA*: immunoglobulin A; *IgG*: immunoglobulin G; *IgM*: immunoglobulin M; *MUC2*: Mucin 2; *B0AT*: solute carrier family 6, member14, *ASCT1*: alanine, serine, cysteine, and threonine transporter; *bo; +AT*: solute carrier family 7, member 9; *GLUT2*: glucose transporter-2; *IFN-γ*: Interferon-gamma.*LAT1*: L type amino acid transporter-1; *PepT1*: peptide transporter-1; *PepT2*:Peptide Transporter 2; *GAPDH*: β-actin, glyceraldehyde 3-phosphate dehydrogenase; *SDHA*: succinate dehydrogenase subunit A.

**References**

Barzegar, S., R. A. Swick, S. K. Kheravii, M. Choct, and S.-B. Wu. 2021. Peroxisome proliferator-activated receptor gamma upregulation and dietary fat levels in laying hens. Poult. Sci. 100:101049.

Du, E., W. Wang, L. Gan, Z. Li, S. Guo, and Y. Guo. 2016. Effects of thymol and carvacrol supplementation on intestinal integrity and immune responses of broiler chickens challenged with *Clostridium perfringens*. J. Anim. Sci. Biotechnol. 7:19.

Fan, X., S. Liu, G. Liu, J. Zhao, H. Jiao, X. Wang, Z. Song, and H. Lin. 2015. Vitamin A deficiency impairs mucin expression and suppresses the mucosal immune function of the respiratory tract in chicks. PLoS ONE. 10:e0139131.

Gharib-Naseri, K., J. C. de Paula Dorigam, K. Doranalli, S. Kheravii, R. A. Swick, M. Choct, and S.-B. Wu. 2020. Modulations of genes related to gut integrity, apoptosis, and immunity underlie the beneficial effects of *Bacillus amyloliquefaciens* CECT 5940 in broilers fed diets with different protein levels in a necrotic enteritis challenge model. J. Anim. Sci. Biotechnol. 11:104.

Gilbert, E. R., H. Li, D. A. Emmerson, K. E. Webb, Jr., and E. A. Wong. 2007. Developmental regulation of nutrient transporter and enzyme mRNA abundance in the small intestine of broilers. Poult. Sci. 86:1739-1753.

Guo, S., D. Liu, X. Zhao, C. Li, and Y. Guo. 2014. Xylanase supplementation of a wheat-based diet improved nutrient digestion and mRNA expression of intestinal nutrient transporters in broiler chickens infected with *Clostridium perfringens*. Poult. Sci. 93:94-103.

Kheravii, S. K., R. A. Swick, M. Choct, and S. B. Wu. 2018. Upregulation of genes encoding digestive enzymes and nutrient transporters in the digestive system of broiler chickens by dietary supplementation of fiber and inclusion of coarse particle size corn. BMC Genomics. 19:208.

Kuchipudi, S. V., M. Tellabati, R. K. Nelli, G. A. White, B. B. Perez, S. Sebastian, M. J. Slomka, S. M. Brookes, I. H. Brown, S. P. Dunham, and K. C. Chang. 2012. 18S rRNA is a reliable normalisation gene for real time PCR based on influenza virus infected cells. Virol J. 9:230.

Kumar, A., S. K. Kheravii, L. Li, and S. B. Wu. 2021. Monoglyceride blend reduces mortality, improves nutrient digestibility, and intestinal health in broilers subjected to clinical necrotic enteritis challenge. Animals. 11:1432.

Lammers, A., W. H. Wieland, L. Kruijt, A. Jansma, T. Straetemans, A. Schots, G. den Hartog, and H. K. Parmentier. 2010. Successive immunoglobulin and cytokine expression in the small intestine of juvenile chicken. Dev. Comp. Immunol. 34:1254-1262.

Li, Z., W. Wang, D. Liu, and Y. Guo. 2018. Effects of *Lactobacillus acidophilus* on the growth performance and intestinal health of broilers challenged with *Clostridium perfringens*. J. Anim. Sci. Biotechnol. 9:25.

Paris, N. E., and E. A. Wong. 2013. Expression of digestive enzymes and nutrient transporters in the intestine of *Eimeria maxima*-infected chickens. Poult. Sci. 92:1331-1335.

Zanu, H. K., C. Keerqin, S. K. Kheravii, N. K. Morgan, S.-B. Wu, M. R. Bedford, and R. A. Swick. 2020. Influence of meat and bone meal, phytase, and antibiotics on broiler chickens challenged with subclinical necrotic enteritis: 1. growth performance, intestinal pH, apparent ileal digestibility, cecal microbiota, and tibial mineralization. Poult. Sci. 99:1540 - 1550.

Zhao, F. Q., Z. W. Zhang, H. D. Yao, L. L. Wang, T. Liu, X. Y. Yu, S. Li, and S. W. Xu. 2013. Effects of cold stress on mRNA expression of immunoglobulin and cytokine in the small intestine of broilers. Res. Vet. Sci. 95:146-155.
