# Supplementary material for: Xylanase and Bacillus subtilis PB6 modulate microbiota and short-chain fatty acid profiles in broilers under necrotic enteritis-challenge
Source: Poult Sci. 2025 Dec 22;105(2):106330. doi: 10.1016/j.psj.2025.106330 (PMC12805170; doi:10.1016/j.psj.2025.106330)
Supplement: Supplementary file 3 [file mmc3.docx]

**Supplementary Table S3**: Effect of xylanase and *B*. *subtilis* PB6 supplementation on d16 caecal bacterial phylum abundance in broilers challenged with necrotic enteritis.

| ^1^Treatments | Firmicutes | Proteobacteria | Actinobacteria | Bacteriodota |
| --- | --- | --- | --- | --- |
| NC | 93.7 | 2.85^b^ | 3.43 | 0.00 |
| CC | 89.8 | 7.09^ab^ | 2.29 | 0.83 |
| Xy | 91.2 | 5.30^ab^ | 2.65 | 0.81 |
| Pb | 91.0 | 5.58^ab^ | 2.70 | 0.76 |
| Xy+Pb | 85.2 | 12.35^a^ | 1.79 | 0.69 |
| ^2^SEM | 2.20 | 2.20 | 0.600 | 0.370 |
| ***P*-value** | 0.082 | 0.032 | 0.465 | 0.449 |

^a-b^ values within a column with no common superscripts differ significantly (*P* < 0.05).

^1^Treatment abbreviations: CC, challenged control; Xy, challenged control+ xylanase (0.03%); Pb, challenged control+ *B*. *subtilis* (0.05%); Xy + Pb, challenged control+ xylanase (0.03%) + *B*. *subtilis* (0.05%); NC, non-challenged control. ^2^SEM: standard error of mean.
