# Supplementary material for: Xylanase and Bacillus subtilis PB6 modulate microbiota and short-chain fatty acid profiles in broilers under necrotic enteritis-challenge
Source: Poult Sci. 2025 Dec 22;105(2):106330. doi: 10.1016/j.psj.2025.106330 (PMC12805170; doi:10.1016/j.psj.2025.106330)
Supplement: Supplementary file 4 [file mmc4.docx]

| ^1^Treatments | Lachnospiraceae | Ruminococcaceae | Lactobacillaceae | Oscillospiraceae | Enterobacteriaceae |
| --- | --- | --- | --- | --- | --- |
| NC | 36.2 | 24.72^a^ | 7.65^b^ | 6.89 | 2.81^b^ |
| CC | 35.5 | 16.87^b^ | 12.16^ab^ | 8.45 | 6.91^ab^ |
| Xy | 31.3 | 12.00^b^ | 21.75^a^ | 6.36 | 5.17^ab^ |
| Pb | 35.1 | 19.34^ab^ | 9.42^ab^ | 9.15 | 5.51^ab^ |
| Xy+Pb | 33.9 | 17.43^b^ | 8.54^ab^ | 7.11 | 12.24^a^ |
| ^2^SEM | 2.60 | 1.80 | 3.30 | 1.10 | 2.10 |
| ***P*-value** | 0.739 | 0.0002 | 0.044 | 0.354 | 0.031 |

**Supplementary Table S4**: Effect of xylanase and *B*. *subtilis* PB6 supplementation on the top-most abundant d16 caecal bacterial family abundance in broilers challenged with necrotic enteritis.

^a-b^ values within a column with no common superscripts differ significantly (*P* < 0.05).

^1^Treatment abbreviations: CC, challenged control; Xy, challenged control+ xylanase (0.03%); Pb, challenged control+ *B*. *subtilis* (0.05%); Xy + Pb, challenged control+ xylanase (0.03%) + *B*. *subtilis* (0.05%); NC, non-challenged control. ^2^SEM: standard error of mean.
