# Supplementary material for: Xylanase and Bacillus subtilis PB6 modulate microbiota and short-chain fatty acid profiles in broilers under necrotic enteritis-challenge
Source: Poult Sci. 2025 Dec 22;105(2):106330. doi: 10.1016/j.psj.2025.106330 (PMC12805170; doi:10.1016/j.psj.2025.106330)
Supplement: Supplementary file 5 [file mmc5.docx]

**Supplementary Table S5**: Effect of xylanase and *B*. *subtilis* PB6 supplementation on the top-most abundant d16 caecal bacterial genus abundance in broilers challenged with necrotic enteritis.

| ^1^Treatments | *Lactobacillus* | *Rumicoccccus_*  *toques* | *Escherichia-Shigella* | *Clostridia-UGC-014* | *Butyricicoccus* | *Subdoligranulum* | *Fecalibacterium* |
| --- | --- | --- | --- | --- | --- | --- | --- |
| NC | 7.65^b^ | 8.17 | 2.81^b^ | 5.03 | 5.78 | 4.78 | 8.80^a^ |
| CC | 12.16^ab^ | 8.21 | 6.90^ab^ | 4.50 | 5.26 | 4.85 | 1.30^b^ |
| Xy | 21.75^a^ | 6.66 | 5.17^ab^ | 8.30 | 4.58 | 2.52 | 1.05^b^ |
| Pb | 9.42^ab^ | 8.59 | 5.51^ab^ | 5.74 | 4.38 | 4.58 | 3.79^ab^ |
| Xy+Pb | 8.54^ab^ | 7.36 | 12.24^a^ | 5.28 | 4.59 | 3.69 | 3.82^ab^ |
| ^2^SEM | 3.40 | 1.00 | 2.10 | 1.50 | 0.800 | 0.700 | 1.50 |
| ***P-value*** | 0.044 | 0.727 | 0.031 | 0.492 | 0.646 | 0.149 | 0.004 |

^a-b^ values within a column with no common superscripts differ significantly (*P* < 0.05).

^1^Treatment abbreviations: CC, challenged control; Xy, challenged control+ xylanase (0.03%); Pb, challenged control+ *B*. *subtilis* (0.05%); Xy + Pb, challenged control+ xylanase (0.03%) + *B*. *subtilis* (0.05%); NC, non-challenged control. ^2^SEM: standard error of mean.
